# Selective forces acting during multi-domain protein evolution: the case of multi-domain globins

**DOI:** 10.1186/s40064-015-1124-2

**Published:** 2015-07-16

**Authors:** Joana Projecto-Garcia, Didier Jollivet, Jean Mary, François H Lallier, Stephen W Schaeffer, Stéphane Hourdez

**Affiliations:** CNRS UMR 7144, Station Biologique de Roscoff, Places Georges Teissier, 29680 Roscoff, France; Laboratoire Adaptation et Diversité en Milieu Marin, UPMC Université Paris 06, Place Georges Teissier, 29680 Roscoff Cedex, France; Department of Biology and Institute of Molecular Evolutionary Genetics, Pennsylvania State University, University Park, PA 16802 USA

**Keywords:** Positive selection, Hemoglobin, Nematodes, Molluscs, Annelids

## Abstract

Multi-domain proteins form the majority of proteins in eukaryotes. During their formation by tandem duplication or gene fusion, new interactions between domains may arise as a result of the structurally-forced proximity of domains. The proper function of the formed proteins likely required the molecular adjustment of these stress zones by specific amino acid replacements, which should be detectable by the molecular signature of selection that governed their changes. We used multi-domain globins from three different invertebrate lineages to investigate the selective forces that acted throughout the evolution of these molecules. In the youngest of these molecules [*Branchipolynoe* scaleworm; original duplication ca. 60 million years (Ma)], we were able to detect some amino acids under positive selection corresponding to the initial duplication event. In older lineages (didomain globin from bivalve mollusks and nematodes), there was no evidence of amino acid positions under positive selection, possibly the result of accumulated non-adaptative mutations since the original duplication event (165 and 245 Ma, respectively). Some amino acids under positive selection were sometimes detected in later branches, either after speciation events, or after the initial duplication event. In *Branchipolynoe*, the position of the amino acids under positive selection on a 3D model suggests some of them are located at the interface between two domains; while others are locate in the heme pocket.

**Electronic supplementary material:**

The online version of this article (doi:10.1186/s40064-015-1124-2) contains supplementary material, which is available to authorized users.

## Background

The increasing number of sequenced genomes has revealed that the fraction of proteins with two or more domains represents up to 70% of the genes in eukaryotes, and tandem repeats in particular represent up to 20% of all sequences in multicellular organisms (for reviews, see Björklund et al. 2005; Han et al. 2007). Both duplication and adaptive evolution have been implicated in the origin and diversity of multi-domain proteins (Vogel et al. 2005). From a limited set of initial domains, the duplication and shuffling of domains has permitted the emergence of numerous and complex proteins with potentially novel functions. Although evolutionary events affecting the domains’ active sites are crucial to determine a proper functioning of the protein, mutations located on the exposed regions of the different domains and at their interface might play an equally important role by affecting folding and interactions between domains (Han et al. 2007). Very few studies have addressed how domains interact to produce the most efficient folding of multi-domain proteins as each domain is usually considered independently, except when the active site is located at their interface (Han et al. 2007; Bhaskara and Srinivasan 2011). Proteins comprised of tandem-repeats of well-known domains could be used to test the mechanisms that are involved in multi-domain proteins evolution to maintain a proper function.

The globin family is one of the most extensively studied protein families, especially in vertebrates. Functional tandem multi-domain globins have so far surprisingly only been found in invertebrates and prokaryotes (Weber and Vinogradov 2001). Invertebrate multi-domain globins have been the target of numerous studies mostly focused on their primary and quaternary structures (Weber and Vinogradov 2001). In addition to the determination of their structure, studies aimed at understanding the evolutionary mechanisms of the origin of the domain repetition. They have shown that unequal crossing-over is the most common mechanism for globin gene duplication (Dewilde et al. 1999; Kato et al. 2001; Naito et al. 1991). Among invertebrates, only nematodes, mollusks, crustaceans, and annelids possess globin subunits with more than one functional protein domain (Weber and Vinogradov 2001) with a great structural diversity.

Nematode hemoglobins (Hbs, i.e. circulating globins) can be single-domain or di-domain (see Weber and Vinogradov 2001 for a review). These latter ones occur in the pseudocoelomic cavity, and assemble into octamers in the pig intestinal parasite *Ascaris suum* (Darawshe et al. 1987; De Baere et al. 1992). The globin gene diversity in nematodes is very large, and intron insertions and gene duplications were frequent in the nematode globin evolutionary history (Blaxter 1993; Hoogewijs et al. 2008; Hunt et al. 2009). However, di-domain Hbs are only known in the gut parasite ascarids *A. suum* and *Pseudoterranova decipiens* (Darawshe et al. 1987; Dixon et al. 1991).

Mollusks can exhibit both hemocyanins and Hbs, the latter being associated with species from hypoxic and fresh-water environments. Mollusk multi-domain Hbs can be found in bivalves and gastropods (see Weber and Vinogradov 2001 for a review). Bivalves of the genus *Barbatia* possess an intracellular di-domain Hb that forms high-molecular weight complexes (Grinich and Terwilliger 1980; Suzuki and Arita 1995; Suzuki et al. 1992). Other bivalves from the families Astartidae and Carditidae have more complex extracellular multi-domain Hb with at least 14–28 domains and gastropods from the family Planorbidae can also have multi-domain Hb with at least 10 domains (Weber and Vinogradov 2001).

In crustaceans, multi-domain Hbs are known in the water fleas *Daphnia* (Dewilde et al. 1999), and *Moina* (Kato et al. 2001), as well as in the brine shrimp *Artemia* (Von Brand et al. 1950), and all are extracellular. *Artemia* represents another example of multiple duplications almost as spectacular as the molluscan multi-domain Hbs. This extracellular Hb possesses nine domains (Manning et al. 1990) and assembles into dimers (Jellie et al. 1996; Matthews et al. 1998).

Typical annelid extracellular globins are renowned and widely studied because of their amazing quaternary structure, which is comprised of a 3.6 MDa hexagonal bilayer (HBL) complex of globins and linker chains (see Weber and Vinogradov 2001 for a review). Annelid extracellular multi-domain globins have so far only been found in *Branchipolynoe*, a hydrothermal-vent endemic genus in the family Polynoidae (Hourdez et al. 1999a). In this genus, the Hb is extracellular, and corresponds to dimers and trimers of tetra-domain globins (Hourdez et al. 1999a). Based on sequence data, Projecto-Garcia et al. (2010) showed that these globins were closely related to single-domain intracellular globins, and were the result of successive tandem gene duplications.

The rare and sporadic occurrence of multi-domain globins in various invertebrate groups indicates multiple origins for these proteins, and suggests a possible selective mechanism for their formation under different environmental circumstances. These are usually linked to the need of organisms to possess complex pigments (not excretable) able to bind a maximum of oxygen molecules when subjected to hypoxia. Functioning as a multi-domain protein potentially requires structural modifications, and the knowledge of the globin tertiary and quaternary structures makes multi-domain globins good candidates to investigate the role of key amino acid replacements in the folding and interdomain interfaces of the protein. In this paper, we studied the selective processes that shaped multi-domain globins, from the initial duplication event to the present-day molecules and which structural modifications these globins underwent until today. We used the annelid *Branchipolynoe*, the blood clam *Barbatia*, and ascarid nematodes, three distinct taxonomic groups in which comparisons between a single-domain and multi-domain genes where possible (occurrence in sister species), to determine whether there was a common mechanism that shaped their structural evolution. These three hemoglobins evolved between 245 and less than 60 million years (Ma) ago, for the ascarids and the polynoids, respectively. Using maximum likelihood approaches, we determined which selective forces were acting during the evolution of the multidomain state and, in particular, identified amino acid positions under positive selection to understand their potential function. These amino acids were placed on a 3D model of each globin to propose structural interpretation of these amino acids.

## Results

Only multi-domain globin sequences for some mollusks, annelids, and nematodes were retrieved from Genbank. Other multidomain globins from invertebrates (arthropods and *Biomphalaria glabrata*) did not meet the selection criteria for the Hb sequences (see “Methods” for details).

**Fig. 1:**
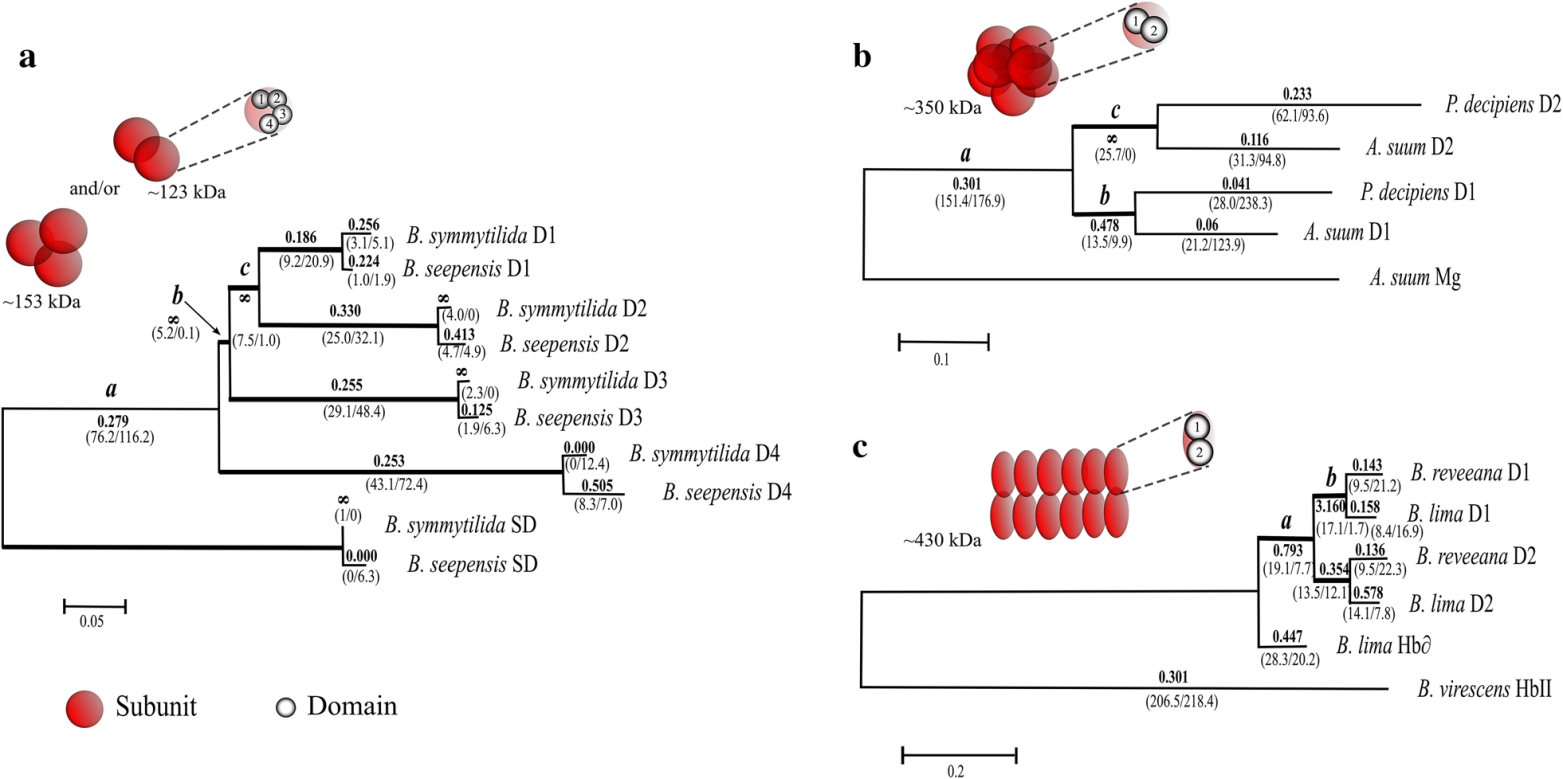
Maximum likelihood trees of the multimeric globin genes from the three groups of metazoans analyzed. **a** *Branchipolynoe* hemoglobin phylogram; **b** NEMATODE globins’ phylogram; **c** *Barbatia* globins’ phylogram. In all cases GTR was used as the nucleotide substitution model. ω values are shown in bold over/under the branches, and N*dN/S*dN ratios are within brackets. *Mg* myoglobin, *SD* single-domain, *D1–D4* domains 1 through 4. *Insets* (adapted from Weber and Vinogradov 2001) represent the quaternary structure of globins to which the correspondent tree is right bellow. Size of the represented assemblies is depicted next to each *inset*.

We were particularly interested in detecting a putative occurrence of codons under positive selection on the branches leading to the formation of the first duplication of the multi-domain globins, but also in all subsequent branches with ω greater than 1 (for which positive selection is suspected), where ω is the ratio of nonsynonymous to synonymous mutations *(d*_N_/*d*_S_) (Fig. 1).

### Selective pressures acting in multi-domain globin evolution

#### Tetra-domain extracellular globin in Branchipolynoe: Positive selection before duplication

Using the single-domain sequences as outgroup, the maximum likelihood tree (Fig. 1a, see “Methods” for details) for the polynoids corresponded to one of the two topologies (topology B) established by Projecto-Garcia et al. (2010). This topology was used as a reference for the following maximum likelihood analyses on coding sequences using the codon model of substitutions.

The likelihood ratio test (LRT = 19.05, df = 16, p = 0.25; Table 1) with the branch model indicates that the ‘free-ratio’ model did not better fit the data than the ‘one-ratio’ model, indicating that the selective pressure did not differ markedly among branches. Most of the evolution of these sequences was characterized by purifying selection (ω = 0.278). This branch model, however, is based on the comparison of the average ω ratio over the whole sequence and if only a few amino acid positions significantly differ, this test may not be able to detect it (Yang 2008). Similarly, the site model, that compares all positions averaged over all the branches, did not reveal any position that had a ω significantly different from the others (Table 1). Overall, most of the positions are under moderate purifying selection (ω = 0.177), although 21% of the positions appear to behave nearly neutrally (ω = 1; Table 1). As selective pressures can also greatly vary between sites on a specific branch, we proceeded with the *branch*-*site model* to test individual amino acid position along each branch. For the branch that leads to the first duplication (branch *a*, Fig. 1a), the likelihood values of the M1a and MA showed that this latter model best fitted the sequence data (LRT = 23.86, df = 2, p = 0.001, Table 1). To determine if this result was a consequence of positive selection or relaxed evolutionary constraints, a comparison was made between the likelihood values of the MA model with the MA_ω=1_ model. This test indicates that the branch corresponding to the first duplication displays evidence of positive selection (LRT = 12.68, df = 2, p = 0.005). Four amino acid residues were identified as being under positive selection with BEB (Bayes Empirical Bayes) values greater than 0.95 (Table 1). Branch *b* and *c* exhibited a *d*_N_/*d*_S_ ratio (ω) value close to infinity (Fig. 1a) but, when tested for positive selection signatures, MA did not better fit the data than M1a (Table 1). This was mainly due to the fact that the two internal branches contained very few mutations, most of them being nonsynonymous mutations (6 and 5 replacements, respectively), and no position had a BEB probability >95%.

**Table 1:** Results from the analyses of positive selection (PAML v 4.7, Yang 2007)

Model	ln*L*	κ	np	Model estimates	LRT (df)	Sites under positive selection (BEB >0.95)
*Branchipolynoe*						
Branch model						
M0	−2,061.60	1.755	19	ω = 0.278		
M1	−2,052.07	1.781	35	0.001 < ω < ∞	19.05^NS^ (16)	
Site model						
M1a ‘nearly neutral’	−2,050.01	1.842	20	ω_0_ = 0.177 (79%) ω_1_ = 1.000 (21%)		
M2a ‘positive selection’	−2,050.01	1.842	22	ω_0_ = 0.177 (79%) ω_1_ = 1.000 (10.8%) ω_2_ = 1.000 (10.2%)	0.00^NS^ (2)	
Branch-site model						
MA branch *a* (duplication)	−2,038.08	1.851	22	ω_0_ = 0.154 (65.6%) ω_1_ = 1.000 (16.3%) ω_2a_ = ∞ (14.5%) ω_2b_ = ∞ (3.6%)	23.86***** (2)	27 V 30A 52Q 65C
MA branch *b* (D4 vs D3 D2 D1)	−2,049.37	1.845	22	ω_0_ = 0.167 (0%) ω_1_ = 1.000 (0%) ω_2a_ = 1.076 (78.5%) ω_2b_ = 1.076 (21.5%)	1.29^NS^	–
MA_branch *c* (D2 D1)	−2,048.87	1.856	22	ω_0_ = 0.167 (0%) ω_1_ = 1.000 (0%) ω_2a_ = 2.6 (79.6%) ω_2b_ = 2.6 (20.4%)	2.28^NS^	–
Nematodes						
Branch model						
M0	−2,153.41	1.455	9	ω = 0.037		
M1	−2146.71	1.313	15	0.029 < ω < ∞	13.4* (6)	
Site model						
M1a ‘nearly neutral’	−2,141.70	1.476	10	ω_0_ = 0.071 (87.3%) ω_1_ = 1.000 (12.7%)		
M2a ‘positive selection’	−2,141.70	1.476	12	ω_0_ = 0.071 (87.3%) ω_1_ = 1.000 (3.0%) ω_2_ = 1.000 (9.7%)	0^NS^	
Branch-site model						
MA branch *a* (duplication)	−2,133.19	1.565	12	ω_0_ = 0.074 (51.2%) ω_1_ = 1.000 (7.2%) ω_2a_ = 1.412 (36.4%) ω_2b_ = 1.412 (5.2%)	17.02***** (2) MA vs MA_ω=1_^NS^	16 sites
MA branch *b* (D1)	−2,135.16	1.407	12	ω_0_ = 0.063 (74.2%) ω_1_ = 1.000 (10.2%) ω_2a_ = ∞ (13.7%) ω_2b_ = ∞ (1.9%)	13.09**** (2)	11A
MA branch *c* (D2)	−2,128.13	1.373	12	ω_0_ = 0.061 (79.1%) ω_1_ = 1.000 (9.5%) ω_2a_ = ∞ (10.2%) ω_2b_ = ∞ (1.2%)	27.14^*****^ (2)	–
*Barbatia*						
Branch model						
M0	−1,989.05	1.398	11	ω = 0.332		
M1	−1,980.29	1.393	19	0.136 < ω < 3.160	17.54** (8)	
Site model						
M1a	−1,967.14	1.514	12	ω_0_ = 0.128 (62%) ω_1_ = 1.000 (34%)		
M2a	−1,956.20	1.649	14	ω_0_ = 0.120 (54.9%) ω_1_ = 1.000 (43.1%) ω_2_ = ∞ (2%)	21.88***** (2)	
Branch-site model						
MA branch *a* (duplication)	−1,961.52	1.572	14	ω_0_ = 0.119 (59.9%) ω_1_ = 1.000 (29.5%) ω_2a_ = ∞ (7.1%) ω_2b_ = ∞ (3.5%)	11.24*** (2)	–
MA branch *b* (D1)	−1,962.64	1.510	14	ω_0_ = 0.117 (62.3%) ω_1_ = 1.000 (31.5%) ω_2a_ = ∞ (4.1%) ω_2b_ = ∞ (2.1%)	9.00** (2)	31G

In the 4 invertebrate groups, the likelihood (*ln*) values correspond to branches leading to various duplications (see Fig. 1a–c) in each phylogram. Selection models implemented in the codeml package, its parameters and associated results of likelihood ratio tests (LRT) are shown (significance threshold: p < 0.05).

*κ* transition/transversion ratio, *np* number of parameters estimated by the model, model estimates: *ω*_*0*_ estimated ω for the category of sites under purifying selection (ω < 1), *ω*_*1*_ estimated ω for sites for the category under neutral evolution (ω ~ 1), *ω*_*2a*_ estimated ω for sites under positive selection in the foreground branches against background branches under purifying selection, *ω*_*2b*_ estimated ω for sites under positive selection in the foreground branches against background branches under neutral evolution, *df* degrees of freedom, *NS* non significant.

* Significant at 0.05; ** significant at 0.025; *** significant at 0.01; **** significant at 0.005; ***** significant at 0.001; BEB >0.95: sites identified by Bayes Empirical Bayes analysis with a posterior probability greater than 95%.

All other internal branches had quite relaxed ω values between 0.255 and 0.33. They were all tested but no signature of positive selection was found. The ω values for the terminal branches were generally characteristic of purifying selection (ω ≪ 1). Interestingly, the exceptions were all on branches leading to *B. symmytilida* sequences (SD, D3, and D2). The results of LRT for these 3 branches showed that M1a model (almost neutral) was systematically chosen against MA with one to two sites positioned in the ω > 1 class. Although not significant (BEB probability ranged between 0.51 and 0.74), these replacements were all found within *B. symmytilida*, suggesting that this newly formed species might have encountered changing conditions since speciation.

Although REL (Random Effects Likelihood, episodic diversifying selection) analysis identified branch *a* as exhibiting the strongest signs of positive selection, the corrected *p* value was not significant (p = 0.13), and the sites identified as under positive selection with PAML were not supported by the MEME analysis.

#### Di-domain nematode Hbs: positive selection in one of the two domains

Based on earlier work (Blaxter 1993; Blaxter et al. 1994; Hunt et al. 2009) we chose the single-domain globins sequences from *A. suum* to serve as outgroup in our tests for selective regimes. In agreement with earlier work and our alignment (Additional file 1: Figure S1) with the sequences of the hemoglobins from *A. suum* and *P. decipiens*, the orthologous sequences of each domain indicated that the duplication occurred before the separation of the two species. We used this unrooted topology (Fig. 1b) for our analysis of selective pressures during the evolution of these di-domain globins.

The ‘free-ratio’ model better fits the sequence data than the ‘one-ratio’ model (LRT = 15.38, df = 6, p = 0.05), indicating that the *d*_N_/*d*_S_ ratios (ω) are heterogeneous among branches. In the branch leading to the nematode multi-domain hemoglobins (branch *a*, Fig. 1b), ω was 0.301, with numerous synonymous and non-synonymous substitutions. For this branch, the ‘site model’ test indicated that the MA model had a significantly greater likelihood value than the M1a model (LRT = 17.02, df = 2, p = 0.001), and BEB analyses identified 16 sites under positive selection with a probability greater than 95%. However, the likelihood for MA was not significantly different from that for MA_ω=1_ (LRT = 0.29, df = 2), indicating that there was no positive selection in the branch leading to the multi-domain nematode Hb or that it was masked by saturation of the phylogenetic signal (accumulation of a very large number of synonymous mutations).

On the branches corresponding to the duplicated domains (branch *b* for domains D1, and branch *c* for domains D2; Fig. 1b), there are more non-synonymous mutations per non-synonymous site than synonymous mutations per synonymous sites, consistent with the expectations for positive selection. For both branches, MA (*branch*-*site model*) better fits the data than M1a (Table 1), indicating heterogeneity of selective pressures among sites. For both of these branches, the LRT between MA and MA_ω=1_ was significant (branch *b*: LRT = 8.31, df = 2, p = 0.025; branch *c*: LRT = 11.51, df = 2, p = 0.005), more specifically indicating the action of positive selection for some amino acid positions. The Bayesian analysis however did not reveal any amino acid position as being significantly under positive selection for domain D2 and only the alanine residue at position 11 was positively selected for D1 (Table 1).

Again, the search for episodic diversifying selection (REL analysis) did highlight branch D1 as well, but the corrected value is not significant, and according to this method the amino acid alanine in position 11 does not appear to be under positive selection.

#### Di-domain intracellular blood clam Hbs

The δ globin chain is considered to be the ancestral single-domain globin sequence for the di-domain of *B. lima* (Suzuki et al. 1996). Our determination of selective forces that acted during the di-domain globin gene evolution was performed with this globin sequence as the outgroup for the phylogeny (Fig. 1c).

The comparison between the ‘one-ratio’ model and the ‘free-ratio’ model indicates that ω is not homogenous among branches (M1 vs. M0, LRT = 17.54, df = 8, p = 0.025). The MA model best fits the data than M1a for branches *a* and *b* (branch-site model; Table 1), suggesting that some amino acids could be under positive selection in these branches. The BEB analysis revealed that residue 31G (located in the AB corner) was under positive selection. None of the other branches exhibited amino acid positions with signature of positive selection.

The REL analysis did not support the findings of the PAML analysis in the blood clam Hbs.

### Location of the amino acid sites under positive selection in a 3D model

The 3D modeling was only performed for the datasets in which we detected potential amino acids under positive selection and a 3D model of the globin subunit was created for only one of the species inside each group of invertebrates.

#### Annelids

**Fig. 2:**
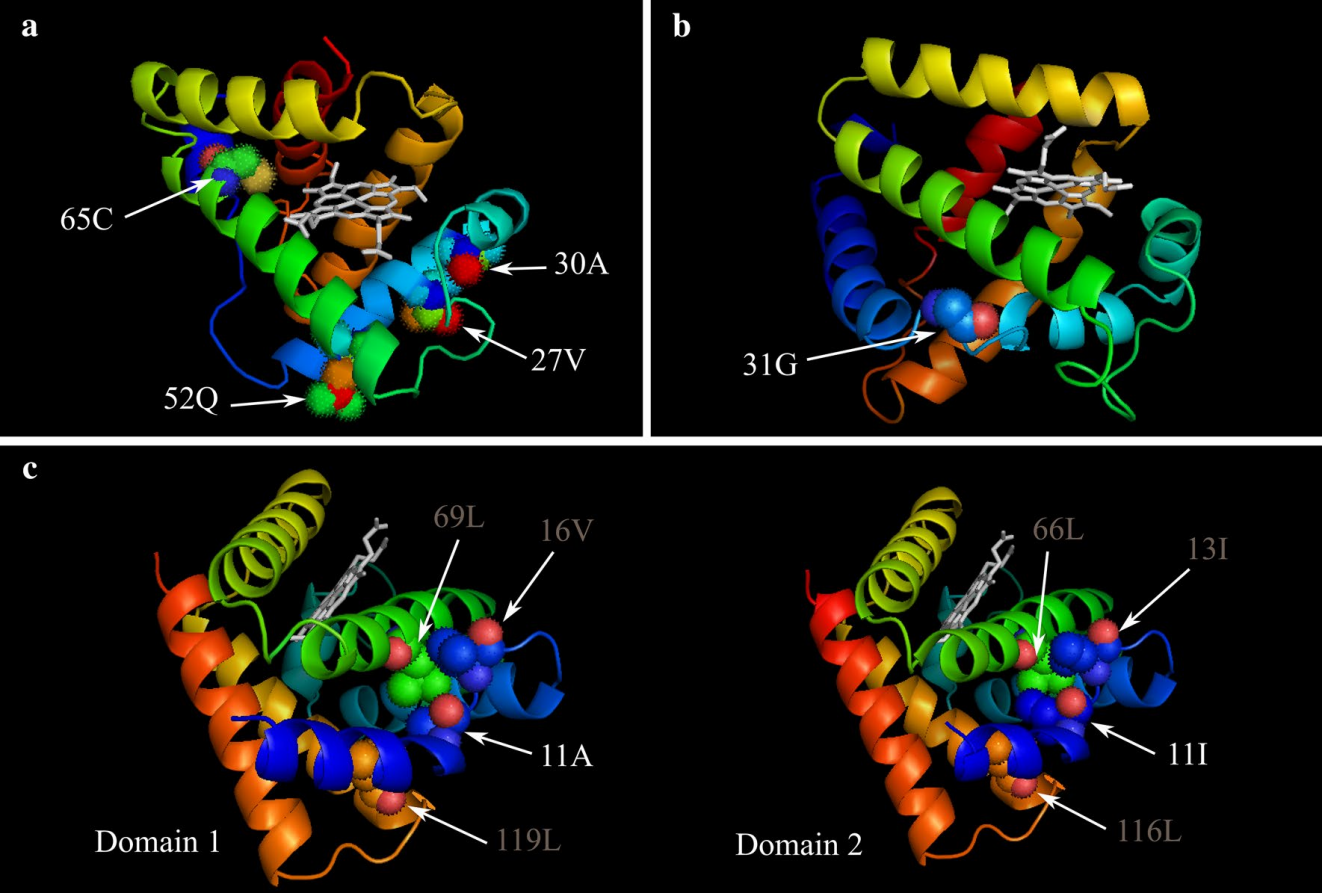
3D models of the globins found in the different species studied, with their heme group. **a** The *B. symmytilida* model was obtained based on *L. terrestris* Hb structure; residues under positive selection are labeled in *white* (27V, 30A, 52Q and 65C). **b** The *B. reveeana* model is based on the tertiary structure of *S. inaequivalis* Hb; the residue under positive selection is shown (31G). **c** *Ascaris* D2 model was built based on the di-domain, D1 crystal (1ash); residue under positive selection on D1 is depicted (11A), name of the residues with possible interactions with amino acid in position 11 are shown in *grey*. A comparative scenario is presented for *Ascaris* D1 and D2 with the interacting residues being in equivalent positions in the protein tertiary structure. Helices are represented in a *color spectrum* depicting helix A in *blue* all through helix H in *red*. Depicted residues are colored by element; OH: *red*; NH: *blue*: C: *helix color*. Heme group is shown in *light grey*. See “Methods” and “Results” sections for detailed information.

The similarity of the four domains, between the 2 species of *Branchipolynoe* ranged between 94 and 97% (Additional file 2: Table S1). Based on this high level of similarity, we only produced the 3D homology model of domain 1 from *B. symmytilida* (Fig. 2a). Three templates were used to produce the 3D models of the Hb D1 from *B. symmytilida*. The first one was the Hb from the polychaete *Glycera dibranchiata* [Protein Data Bank (PDB) 1HBG] as this is one of the closest species with a globin crystal. The second corresponds to another annelid species, *Lumbricus terrestris* (PDB 1X9F), and the third template was Hb from the cestode *Gasterophilus intestinalis* (PDB 2C0K), the closest sequence automatically chosen by the SWISS-MODEL server. Out of the three models, the one that we considered the best was the model based on the *Lumbricus* Hb sequence (based on the ANOLEA and GROMOS graphics, and by comparing the QMEAN descriptors—see details in “Methods”). Although the model based on *G. intestinalis* had a smaller QMEAN value (the lower the predicted energy, the better the model) the *Lumbricus*-based model exhibited smaller errors in the parts of the quaternary structure where the residues under positive selection are located.

**Fig. 3:**
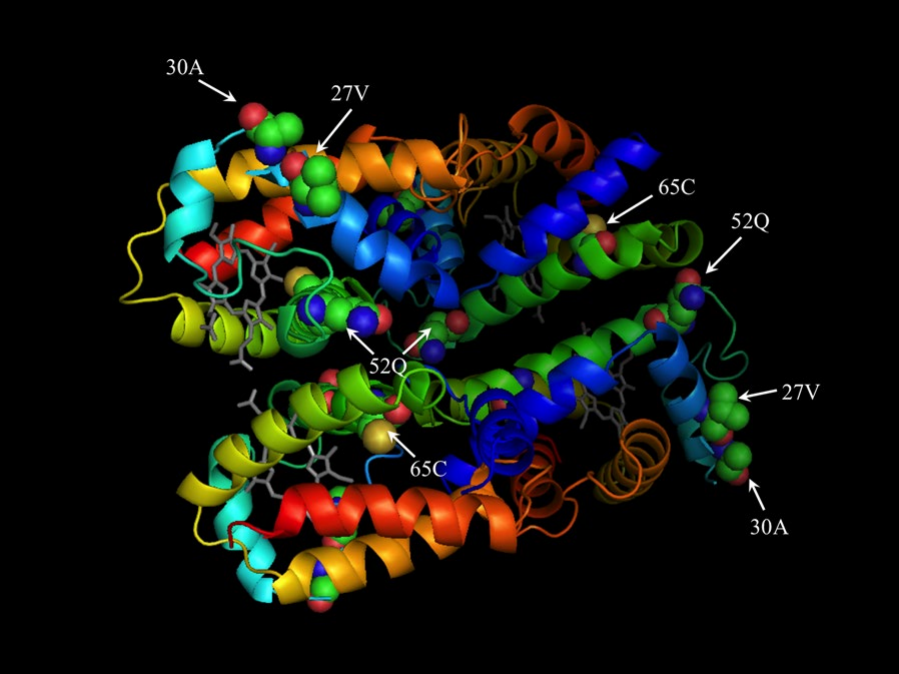
Theoretical 3D model of an annelid tetramer based on *B. symmytilida* D1 sequence and the crystal structure of *Lumbricus terrestris* (1X9F). Amino acids identified as potentially under positive selection are highlighted. 52Q appears to be in an area where it could participate to the formation of an oligomer. Depicted residues are colored by element; OH: *red*; NH: *blue*: C: *helix color*. Heme group is shown in *grey*. Model constructed with Modeller (Mod9v13, Eswar et al. 2006).

The cysteine in position 65 (65C) is located in the far end of the heme pocket and is surrounded by a slightly hydrophilic and polar cluster (Additional file 3: Figure S2). The remaining amino acids identified by BEB analysis under positive selection are located in the B helix (27V and 30A), close to the DE corner, and on the E helix (52Q) (Fig. 2a). The substitutions of histidine for a valine and glutamine for an alanine on the B helix from the single-domain to the tetra-domain produce a more hydrophobic exposed surface of the protein in the area. In the vicinity of the DE corner, the replacement of an alanine by a glutamine in position 52 (E5), on the contrary, creates a more hydrophilic surface. When using *L. terrestris* crystal structure to model the theoretical tetramer based on *B. symmytilida* D1 sequence, it becomes clear that this same residue (52Q) occupies a central position in the tetramer (Fig. 3).

#### Nematodes

A crystal structure was already available in the PDB for *Ascaris* hemoglobin D1 (1ASH), and this was the chosen template to model D2 (SWISS-MODEL server). We identified a single residue in domain D1 as being under positive selection (A11) through BEB analysis. This amino acid is located on the loop between helices A and B (Fig. 2c), and points toward the core of the protein, suggesting that the substitution could affect the stability of helix A. This Ala replaces an Ile and, even though both are hydrophobic, the latter is larger and strongly hydrophobic. The Ile forms closer interactions with other hydrophobic amino acids, in particular on helix E (Leu in position 66 for D2 and 69 in D1) and, to a lesser extent, with the end of helix G (Leu in position 119 on D1 and 116 in D2; Fig. 2c). Ile13 (in D2) and Val16 (in D1) are also likely to participate in this hydrophobic cluster. The Ala in position 11 could allow a looser interaction with the hydrophobic cluster located in this region, possibly resulting in a more flexible region of the protein.

#### Globin domain of blood clams

The similarity between each hemoglobin domain of *B. reeveana* and *B. lima* was ~91% for domain 1 and ~88% for domain 2 (Additional file 2: Table S1). The high level of identity allowed us to only use one of the two domains from one species to produce the 3D model. The model of D1 from *B. reeveana* Hb was automatically obtained through the homology of the quaternary structure of the hemoglobin from *Scapharca inaequivalvis* (PDB 1SCT), the closest sequence with a crystal structure currently available. *S. inaequivalis* is a bivalve from the family Arcidae, specialized in anoxic and sulfidic environments (de Zwaan et al. 1995). The packing quality of the model (ANOLEA) and the empirical force field (GROMOS) were overall good (more negative energy values), including the area were we found the residue under positive selection. The residue 31G, identified as being under positive selection by BEB analysis, is located in the corner between helices A and B (Fig. 2b). This uncharged weak polar residue is surrounded by a strong hydrophilic polar cluster (Additional file 4: Figure S3).

## Discussion

The basic tertiary structure of globins—the globin fold—is highly conserved in all domains of life (Weber and Vinogradov 2001). Although vertebrate circulating hemoglobins always exhibit a tetrameric structure involving two types of subunits (the iconic α_2_β_2_ structure), invertebrate hemoglobins have a great diversity of quaternary structures, from dimers to a staggering 180-subunit assemblage (Weber and Vinogradov 2001; Royer et al. 2005). Despite this great structural diversity, all invertebrate allosteric hemoglobins possess a similar basic dimeric unit, termed “EF dimers” because of the extensive implication of amino acid residues from helices E and F at the interface between the two subunits (Royer et al. 2001). Further assembly of subunits is obtained by interaction of these basic EF dimers. The high level of conservation of the basic globin folding and inter-subunit interactions allows us to make predictions about the role that the amino acids under positive selection could play. This study of three very diverse taxonomic lineages allowed us to test whether the predicted structural constraints produced similar selective response (adaptive structural convergence) when facing challenging environmental conditions (in this case hypoxia). Positive selection could also affect the function of each domain differentially, leading to domains with different, specialized characteristics (neofunctionalization).

In all our analyses, the findings of the PAML analyses were not significantly supported by the REL analyses, although the branches identified by the former were also the ones exhibiting the strongest signal of episodic diversifying selection in the latter type of analyses. The following discussion is therefore centered on the PAML results. Although care should be taken when using d_N_/d_S_ analyses and BEB determination of amino acids under positive selection (Zhang et al. 2005), the identified residues occupy positions for which structural and/or functional predictions can be made (inter- and intra-subunit interactions, affinity for oxygen).

### Annelida: a recent and special multi-domain Hb

Multi-domains Hbs in annelids are so far only known in species of the genus *Branchipolynoe*, that lives commensally with mussels from hydrothermal vents and cold seeps (Hourdez et al. 1999a). These hemoglobins evolved recently (ca. 60 Ma ago) in this group of scaleworms, and were obtained by tandem duplication of a single-domain gene coding for a myoglobin-like protein (Projecto-Garcia et al. 2010). We detected that diversifying selection has impacted the primary structure of the *Branchipolynoe* Hbs domains before the duplication process, and this *d*_N_/*d*_S_ heterogeneity was due to positive selection that acted in the branch corresponding to the initial duplication event. This suggests that amino acid changes were important for the formation of the multi-domain globin, for structural and/or functional reasons. The other internal branches of the domains’ phylogeny all had ω ≪ 1, suggesting that after the positive selection at the first duplication event, purifying selection acted to conserve the modified amino acids, and that no further modification was necessary to allow the functioning of the globin with more than two domains. Some of the terminal branches for *B. symmytilida* also display high ω values. This could indicate specialization of this species tetra-domain Hb to slightly different environmental conditions. In the evolution of the *Branchipolynoe* species the Pacific *B. symmytilida*, living around vents, seems to derive from a cold seep ancestor, morphologically close to the Atlantic *B. seepensis*, whose actual ‘cold seep’ intermediates are presently located on both parts of the Panama Isthmus. Alternately, there are two types of hemoglobins in *Branchipolynoe*, one forms dimers and the second trimers (Hourdez et al. 1999a). It is possible the sequences used here are paralogs rather than orthologs. If this is the case, our results could be indicative of subfunctionalization after tetra-domain gene duplication. The BEB test did not detect specific amino acids under positive selection on the terminal branches. However, the branch-site model and BEB analysis are probably not powerful enough when the number of nucleotide substitutions in the foreground branch is small (Nozawa et al. 2009).

Duplication events are common in eukaryote lineages, producing gene families, with paralogs either carrying on the function of the parent gene or giving rise to novel functions (by neofunctionalization or subfunctionalization; Britten and Kohne 1968; Duboule and Wilkins 1998; Voordeckers et al. 2012). Assuming an intracellular globin gene as the parental gene for the tetra-domain lineage (Projecto-Garcia et al. 2010), the paralogs seem to have functions similar to that of their ancestor. *Branchipolynoe* tetra-domain globins possess high oxygen affinity and a low cooperativity (Hourdez et al. 1999b), both characteristics reminiscent of myoglobin function, suggesting that the amino acid changes affect the structure rather than the function of the multi-domain hemoglobin. *Branchipolynoe* extracellular hemoglobins also form multimers (Hourdez et al. 1999a) while myoglobins are usually monomeric. The observed changes could then be related to this capacity to form oligomers.

Some of the amino acids under positive selection are located in the B helix (27 V and 30A), close to the DE corner (Fig. 2a). Because of their position and their hydrophobicity these amino acids are likely to be involved in the interface between two domains. In multi-domain proteins the residues found in interface regions are mostly non-polar to allow stable interaction regions (Bhaskara and Srinivasan 2011) and in both positions, 27 and 30, polar residues were substituted by non-polar derived amino acids, H → V and Q → A, respectively (Additional file 3: Figure S2). The glutamine in position 52 (52Q) is in the E helix, and could be involved in the formation of oligomers (Fig. 3), which usually involves the interaction of the helices E and F between subunits (Royer et al. 2005). In this position the ancestral non-polar residue, an alanine, was substituted by a glutamine, a polar amino acid. This could be advantageous as, based on the probable configuration of the multi-domain folding (Fig. 3), this glutamine can establish a hydrogen bond with the polar serine (located early in the F helix). The 65C is located in the far end of the heme pocket, but the distance to the distal heme is too large to affect oxygen-binding properties. This cysteine is particularly interesting as it is under positive selection in some extracellular globins from other annelids that live in sulfidic environments (Bailly et al. 2003). This amino acid, located at the exact same position, was thought to be involved in the reversible binding of sulfide in *Riftia* (Zal et al. 1998) but this mechanism of sulfide binding has since been challenged (Flores et al. 2005; Flores and Hourdez 2006). Its occurrence in a lineage of extracellular globins distinct from the typical HBL-Hb globins (and its absence in the single-domain intracellular globins) suggests that it is the result of convergent evolution. Its function remains unclear but its presence in an extracellular hemoglobin from a species that lives under sulfidic conditions points towards a possible function in protecting the heme from reacting with sulfide (which would form sulfhemoglobin, no longer capable of reversibly binding oxygen).

### Nematode Hb

Nematode globins have a very rich evolutionary history (Blaxter 1993; Hoogewijs et al. 2008; Hunt et al. 2009). The duplication of this multi-domain Hb gene most likely happened in the common ancestor of *Ascaris* and *Pseudoterranova*, because each domain clusters together (Blaxter 1993). In both species the di-domain Hbs exhibit a high oxygen affinity, and share amino acid residues in the heme pocket that are thought to be responsible for the slow release of oxygen from the heme (Gibson et al. 1993).

These are the highest oxygen affinities measured in animal globins, and we therefore expected to find signatures of positive selection in the lineage leading to the duplicated nematode Hb gene. The fact that we did not find positive selection in the branch between the single- and the di-domain globins could be due to two reasons: (1) the time of the duplication (by unequal crossing-over) is estimated to be 245 Ma in *P. decipiens* (Dixon et al.^1992^) (and we can assume the same time for *A. suum* Hb gene duplication based on their orthology), a time probably sufficient to erase signatures of selection because of the accumulation of synonymous substitutions (Yang 2008); and (2) the fact that the residues (B10Y and E7Q) that are thought to be responsible for the high oxygen affinity in the di-domains (De Baere et al. 1994) are also present in the myoglobin of *A. suum* (Blaxter et al. 1994), used in these study as an outgroup for our topology. However, Blaxter et al. (1994) suggest that these residues may not be the only requirement for high oxygen affinity, other amino acids possibly affecting the distance between the B10Y residue and the di-oxygen bound to the heme might be also responsible.

As in annelids, it seems that the amino acid we identified as being under positive selection more likely has a structural rather than functional effect. The residue 11A could be responsible for a greater flexibility of the protein, in particular by affecting the stability of the E-helix. This amino acid could indeed form weaker hydrophobic bonds with residues on the A–B corner and the E-helix. This could also be important during O_2_ association/dissociation processes and, according to Kloek (1993), D1 does exhibit a faster O_2_ dissociation rate than D2.

It is possible that the most important difference between a single-domain and a di-domain Hb, in *Ascaris*, is the fact that the di-domain Hb can form octamers (Darawshe et al. 1987; De Baere et al. 1992). In *Ascaris lumbricoides*, the octameric structure is obtained by the interaction of a highly charged C-terminus tail that acts as an intra-molecular chaperone, and the interaction is then stabilized by interactions between globin folds, especially by the leucine residue in position 15 (Minning and Goldberg 1998).

### Molluskan Hbs

Mollusks have remarkable multi-domain Hbs. Not only are their genes made of more that one domain, the number of repeats of these domains is of a magnitude only found in other protein families in vertebrates (Björklund et al. 2005). The di-domain Hb from *Barbatia* did not reach a spectacular state of duplication, it is, however, an intracellular Hb, and in this category its molecular weight (~430 kDa, result of ~35 kDa subunits assemblage in dodecamers) remains unrivaled (Grinich and Terwilliger 1980; Grinich et al. 1986). The D1 lineage from *Barbatia* exhibits a ω greater than 1, in sharp contrast with the D2 lineage with an ω ~ 0, highlighting structural (and possibly functional) changes on D1, while D2 experiences slight purifying selection (ω = 0.354). The residue 31G (AB corner) was identified as being under positive selection in the D1 lineage. This corner, in particular, is extensively involved in contacts used to assemble the dimers of HbII into tetramers in *Scapharca*, another arcid clam (Royer et al. 1995). In *Barbatia*, it is mainly a hydrophilic region (Additional file 4: Figure S3): the residue occupying this position is a glycine (weakly polar) in D1 and a lysine (polar) in D2. The very different size of the side chains is likely to affect the oligomerization of these subunits, and glycine would probably have a stabilizing effect in this interaction region through its lower hydrophilic character. Lysine in this position is found not only in the D2 of *B. lima* and *B. reveeana*, but also with the outgroup sequences, Hb δ from *B. lima* and HbII from *B. virescens*, supporting its importance in comparison with the same site in D1. The presence of a short interdomain peptide (termed ‘linker’) in *Barbatia* (Naito et al. 1991) may also allow a greater flexibility between the two domains, and thereby relax the pressure that the too-close setting of the tandem duplication represents. Only the crystallization of the native 430 kDa component of *Barbatia* hemoglobin or its 220 kDa dissociation product (Grinich and Terwilliger 1980) could shed light of the amino acids involved in actual interactions, and the potential effect of the linker.

## Conclusion

### Multi-domain proteins: selection and folding

Our analyses on all three datasets of multi-domain proteins showed that most amino acid positions are under moderate purifying selection (ω < 1), some appear to behave neutrally (ω = 1), and very few have been under positive selection. These latter however seem to be mainly located in positions where they can affect the structure rather than the function of the proteins.

Based on genome comparisons (from prokaryotes and eukaryotes), it seems that the occurrence of a single duplication event is much more common (giving rise to di-domains) in protein genes than several duplication events (Apic et al. 2001; Björklund et al. 2005), except for protein genes that were already multi-domain. In this case the replication of several domains at once is usually the norm, producing domain repeats (Björklund et al. 2005). As for other metazoans, the domain duplication in *Branchipolynoe* could be an internal response to a specific environmental constraint (Apic et al. 2001). In *Branchipolynoe*’s case, the low levels of oxygen (hypoxia) that result from the mixing of the anoxic hydrothermal vent fluid with the deep-sea water could be the environmental constraint that favored the appearance of the multi-domain hemoglobin (Hourdez et al. 1999b). Hypoxia may also be the constraint responsible for the multi-domain hemoglobin evolution for the two other taxa: the two nematodes are gut parasites of vertebrates, and the bivalve is found in low oxygen marine environments.

All the discussed multi-domain Hbs have different evolutionary single-domain points of origin, the first duplication occurred at very different times: estimated to be about 245 Ma ago for the nematodes (Dixon et al. 1992), before the speciation process that led to the emergence of *Barbatia* species about 165 Ma ago (Plazzi and Passamonti 2010, and less than 60 Ma ago for *Branchipolynoe*, as the duplication event occurred after the scale-worm radiation (Projecto-Garcia et al. 2010). This will undoubtedly have a profound blurring effect on the signal of selective pressures in the deeper branches of the gene phylogenies, as more synonymous substitutions will have accumulated in the older lineages and the positive selection signal attenuated.

The proper folding of multi-domain proteins (and in particular tandem duplicated ones) can be problematic if the contact areas are not sufficiently different (Han et al. 2007). As a result, contact areas in multi-domain proteins usually consist of less similar domains (Han et al. 2007). The residues in *Branchipolynoe* Hb, near the DE corner, in the B and E helices are serious candidates to achieve a proper folding of the tetra-domain Hb, as well as polymerization to form the dimers and trimers of tetra-domain globins found in *Branchipolynoe* (Hourdez et al. 1999a). To date, there are no known interactions of these Hbs with other proteins and the observed changes are unlikely to correspond to intermolecular coevolution. However, this possibility cannot be ignored for other multi-domain proteins.

In invertebrate multi-domain globins, the polymerization and cooperativity depend mostly on the E–F dimer structure (interactions between E and F helices from different subunits) (Royer et al. 2001; Riggs 1998). Bigger structures, opposed to a myoglobin or single-domain Hb, may be important to avoid their accidental elimination or to maintain a low oncotic pressure while increasing oxygen carrying capacity.

### Support of predictive models of interdomain interactions

These observations raise the interesting possibility that this approach could be used as a method that points towards amino acids found at the interface between two domains of a protein. In particular, it could be used in support of predictive models for intermolecular contacts. It is indeed sometimes difficult to obtain crystals of multidomain proteins, and research must rely on isolated domains structures. The approach we used could be used in virtually all mutidomain proteins, and help identify interaction areas if these are not known from crystal structures.

## Methods

### Sequence retrieval

Multi-domain globin sequences for mollusks, annelids, and nematodes were retrieved from Genbank. The selection criteria for the Hb sequences were to find a phylogenetic group where one could find a multi-domain Hb, and that has a single-domain globin (or a closely related taxon with this single-domain globin) for comparison. For arthropods, as well as for some mollusks, this was not possible. As a result, we only used globin sequences from *Barbatia reveeana* (M73328), *B. lima* (delta chain Hb: D63932, alpha chain Hb: D63933, beta chain Hb: D63934, di-domain Hb: D58417), *B. virescens* (chain II Hb: D58416). Although sequences for the snail *Biomphalaria glabrata* are available, a reconstruction of the phylogeny of the 13 globin domains yielded very low confidence values for most of the deep branches (data not shown). This could greatly affect our confidence in the reconstruction of ancestral sequences, and this dataset was therefore cast aside. For nematodes: we retrieved sequences from *Ascaris suum* (myoglobin: U17337, di-domain Hb: L03351), *Pseudoterranova decipiens* (di-domain Hb: M63298) and the single-domain globins from *Caenorhabditis elegans* (Z18264) and *Trichostrongylus colubriformis* (M63263). For annelids we used sequences from two species of the scale-worm genus *Branchipolynoe*; *B. symmytilida* and *B. seepensis* (tetra-domain globins GQ360749–GQ360756, and their single-domain globins GQ360757–GQ360758, respectively).

### Phylogenetic analyses

For all sequence datasets, multiple nucleotide and amino acid sequence alignments were performed with the multiple sequence alignment algorithm MUSCLE (Edgar 2004, part of software Geneious Pro 5.3.6, created by Biomatters). During sequence alignment optimization, we aimed at minimizing the number of indels, and because we were dealing with coding sequences, the nucleotide alignment was constrained by the amino acid sequences alignment. Although the number of sequences was small in each taxonomic group, we compared the alignments obtained with MUSCLE and submitted the raw amino acid sequences to the GUIDANCE filter (Penn et al. 2010), using the alignment algorithm MAFFT (Katoh et al. 2005). MUSCLE and MAFFT produced very similar outputs and we therefore chose the alignments produced by the former (Additional file 1: Figure S1, Additional file 3: Figure S2, Additional file 4: Figure S3). GUIDANCE provided us alignment scores and regions of the alignments that were not well supported (Additional file 1: Figure S1, Additional file 3: Figure S2, Additional file 4: Figure S3). These regions were removed from further calculations in the PAML software.

#### Tree topologies

For each group of invertebrate we aimed at testing the evolutionary mechanism underlying the transition from single- to multi-domain proteins. Although phylogenies including the sequences we studied have been published earlier (*Barbatia*: Suzuki et al. 1996, nematodes: Blaxter et al. 1994), these often included numerous other sequences. Our topologies only included the orthologs of the multi-domain Hb from each group and, for comparison, the nearest single-domain globin sequence available for one of the genera that had a multi-domain Hb. Working topologies were generated by maximum likelihood (1,000 bootstrap replications), using MEGA 5 (Nei and Kumar 2000; Tamura et al. 2011) for each of the limited datasets. The best substitution model for each dataset was determined beforehand with jModelTest (Posada 2008) and in all cases the GTR model was selected.

#### Analyses of selection regimes

The determination of the selection regimes that acted in the evolution of the multi-domain globins was performed by maximum likelihood analyses on the coding sequences, using the software PAML 4.7 (Yang 1997, 2007). These analyses were also performed with the MEME and REL methods (Murrell et al. 2012), implemented on the Datamonkey.org webserver, to confirm the identification of the sites under selection. Only the PAML method is detailed here, but the methods in Datamonkey also use maximum likelihood algorithms.

The PAML approach uses the codon model of substitutions developed by Goldman and Yang (1994) implemented in Codeml (Nielsen and Yang 1998; Yang 1998; Yang and Nielsen 2002). When using codeml, we chose the mode ‘clean data = 1’ allowing us to remove all the sites with ambiguous characters and alignment gaps (Yang 1997).

Unrooted tree topologies were used with the condition that single-domain globins or myoglobins form a reciprocal monophyly with respect to the multi-domain globin sequences. At first two branch models, a ‘one-ratio’ and a ‘free-ratio’ models, were compared with a likelihood ratio test (LRT), to test whether ω was homogenous among all lineages (H_0_ = ‘one-ratio’ model best fits the data, H_1_ = ‘free-ratio’ model best fits the data). The LRT is expected to have a χ^2^ distribution, and the result can then be evaluated in a χ^2^ table, with the number of degrees of freedom equal to the difference in the number of parameters between the models (Yang 1998). When H_0_ was rejected (*i.e.* ω not homogenous amongst lineages), we used a *branch*-*site model* (Yang and Nielsen 2002; Zhang et al. 2005), where ω can vary among codon sites in a foreground lineage when compared to the whole tree (background sites), allowing the identification of amino acid sites under positive selection in that specific branch (Yang and Nielsen 2002). H_0_ then corresponds to the fitting of the dataset to a nearly-neutral evolution model (M1a in which ω can be classified into 2 classes: 0 < ω < 1 or ω = 1), and H_1_ to the fitting to a model that assumes positive selection among sites of the foreground lineage (Model A (MA) in which ω can be classified into 3 classes: 0 < ω < 1, ω = 1 and ω > 1). These two models were compared by LRT and when the MA best fitted the data, we proceeded to one additional comparison; between MA and MA_ω=1_ (where ω is forced to be 1 in the third class) to distinguish relaxed selective constraints from positive selection (Yang and Nielsen 2002; Wong et al. 2004). When this LRT was significant, suggesting the presence of sites under positive selection, the identification of these sites was done by Bayesian analysis, and only sites with a posterior probability greater than 95% were conserved (Yang 2008). We used the Bayes Empirical Bayes (BEB) analysis preformed by the Codeml package. That method accounts for the sampling errors in maximum likelihood estimates of model parameters (compared to the Naive Empirical Bayes analysis), which could be important in small datasets like ours (Yang et al. 2005).

### Sequence-based protein 3D modeling

The 3D protein models were obtained through the automated protein structure homology-modeling server SWISS-MODEL (Arnold et al. 2006; Kiefer et al. 2009; Peitsch 1995). The quality of the models was assessed through the values of the atomic empirical mean force potential or packing quality of the model (ANOLEA, Melo and Feytmans 1998) and the empirical force field (GROMOS, Eisenberg et al. 1997). These tools measure the goodness of fit between the quaternary structure of the amino acid sequence of interest and the crystal reference used as template. In both cases the more negative the energy values, the more favorable the energy environment. A QMEAN (Benkert et al. 2008) was also used to judge the fitness of the model. This mean is a composite scoring function that accounts at least for the error of residue allocation, comparison with other known 3D models and hypothetical performance of the model through X-ray analyses (cf. SWISS-PROT website). MacPyMOL (PyMol Version 1.3 2010) was used to visualize and edit the produced model, in particular to highlight amino acid residues of interest.

## Additional files

Additional file 1:
**Figure S1.** Alignment of the single-domain and the di-domain globin sequences from the two nematode species. Overall sequence identity (red = low identity, green = high identity, the height of blocks is proportional to the percentage of identity) is represented above the sequences. Please refer to the legend in Figure S2 for the color codes in nucleotide identity and residue hydrophobicity. These sequences were generated from mRNA and for this reason exon limits are not depicted. Alignments were obtained by MUSCLE and submitted to the GUIDANCE filter (more details in the “Methods” section). The regions of the alignments that were not well supported by the filter were removed (shaded areas) from further phylogenetic analyses. Asuum: *Ascaris suum*, Pdec: *Pseudoterranova decipiens*, Mg: myoglobin, D: domain. Additional file 2:
**Table S1.** Identity between orthologs domains (amino acid sequences) from the three analyzed groups of multidomain invertebrate Hbs. Additional file 3:
**Figure S2.** Alignment of the single and tetra-domain globin sequences from the two Polynoidae species. The initial methionine was removed. Overall sequence identity (red = low identity, green = high identity, the height of blocks is proportional to the percentage of identity) is represented above the sequences. The limits of the exons are indicated by the grey bars. Alignments were obtained by MUSCLE and submitted to the GUIDANCE filter (more details in the “Methods” section). The regions of the alignments that were not well supported by the filter were removed (shaded areas) from further phylogenetic analyses. Bsy: *Branchipolynoe symmytilida*, Bse: *B. seepensis*, D: domain, SD: single-domain. Additional file 4:
**Figure S3.** Alignment of the single-domain and the di-domain globin sequences from the mollusk species. Overall sequence identity (red = low identity, green = high identity, the height of blocks is proportional to the percentage of identity) is represented above the sequences. Please refer to the legend in Figure S2 for the color codes in nucleotide identity and residue hydrophobicity. These sequences were generated from mRNA and for this reason exon limits are not depicted. Alignments were obtained by MUSCLE and submitted to the GUIDANCE filter (more details in the “Methods” section). The regions of the alignments that were not well supported by the filter were removed (shaded areas) from further phylogenetic analyses. Bvire: *Barbatia virecens*, Blima: *Barbatia lima*, Brev: *B. reveeana*, Hb: hemoglobin, D: domain.

## Abbreviations

Ma: million years
Hb: hemoglobin
Mg: myoglobin
SD: single-domain
D: domain
MDa: mega daltons
HBL: exagonal bilayer
LRT: likelihood ratio test
BEB: Bayes Empirical Bayes
REL: random effects likelihood
MEME: mixed effects model of evolution
PAML: phylogenetic analysis by maximum likelihood
PDB: protein data bank
ω: ratio of nonsynonymous to synonymous mutations (*d*_N_/*d*_S_)
*d*_N_: rate of nonsynonymous substitutions
*d*_S_: rate of synonymous substitutions
