# Supplementary figures and images for: Selective forces acting during multi-domain protein evolution: the case of multi-domain globins
Source: Springerplus. 2015 Jul 16;4:354. doi: 10.1186/s40064-015-1124-2 (PMC4503718; doi:10.1186/s40064-015-1124-2)

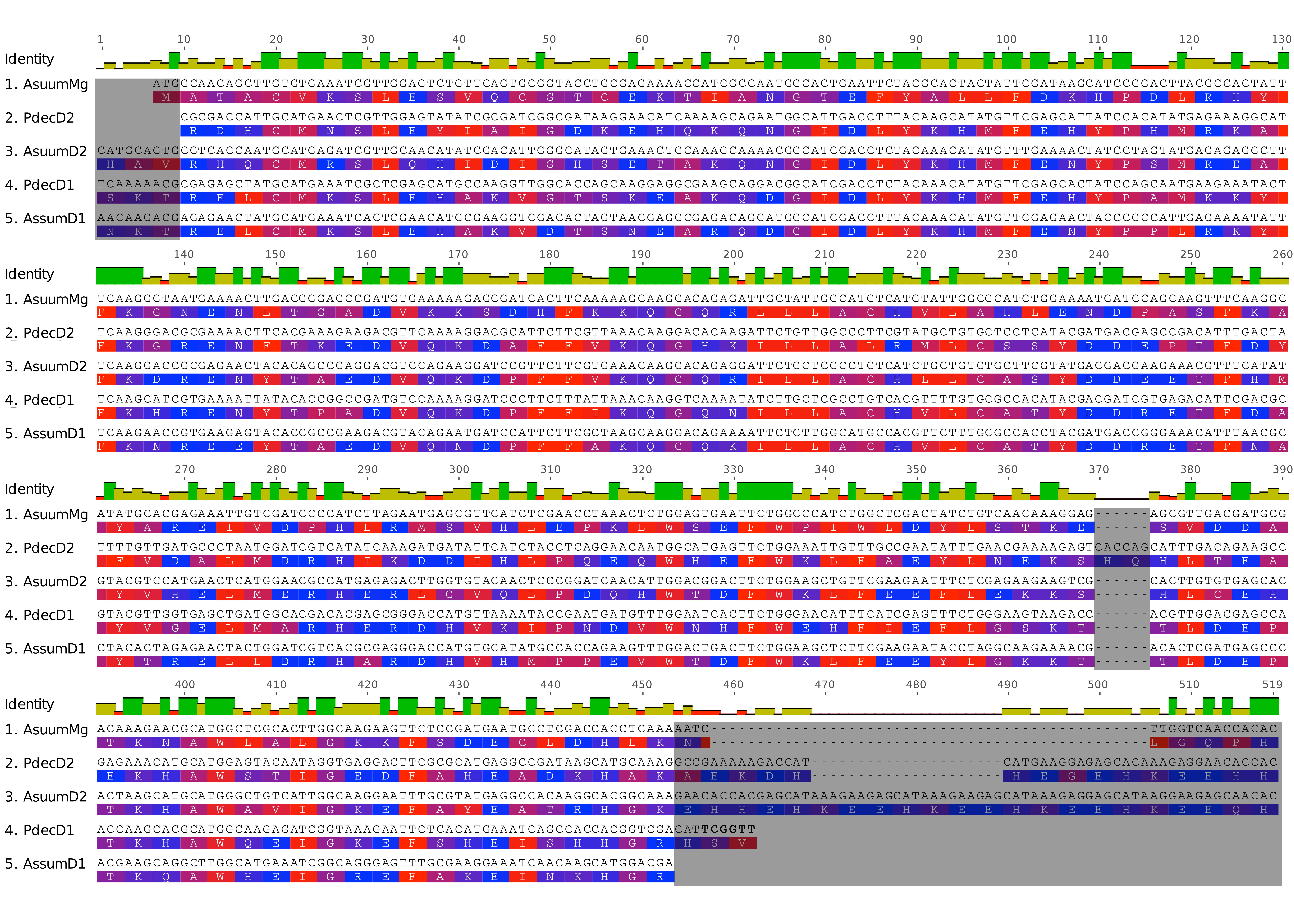

Supplement: Additional file 1: — Figure S1. Alignment of the single-domain and the di-domain globin sequences from the two nematode species. Overall sequence identity (red = low identity, green = high identity, the height of blocks is proportional to the percentage of identity) is represented above the sequences. Please refer to the legend in Figure S2 for the color codes in nucleotide identity and residue hydrophobicity. These sequences were generated from mRNA and for this reason exon limits are not depicted. Alignments were obtained by MUSCLE and submitted to the GUIDANCE filter (more details in the “Methods” section). The regions of the alignments that were not well supported by the filter were removed (shaded areas) from further phylogenetic analyses. Asuum: Ascaris suum, Pdec: Pseudoterranova decipiens, Mg: myoglobin, D: domain. [file 40064_2015_1124_MOESM1_ESM.png]

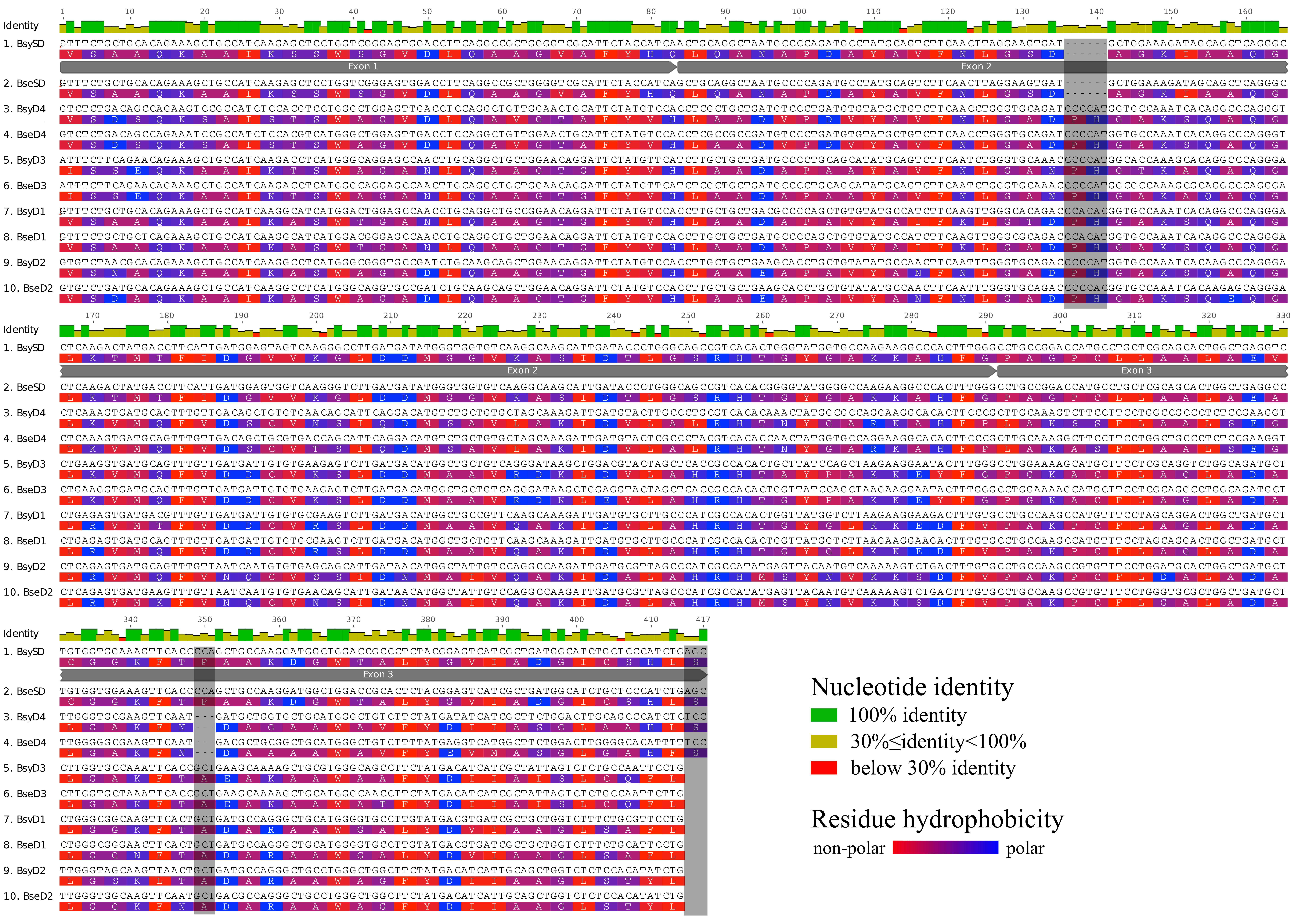

Supplement: Additional file 3: — Figure S2. Alignment of the single and tetra-domain globin sequences from the two Polynoidae species. The initial methionine was removed. Overall sequence identity (red = low identity, green = high identity, the height of blocks is proportional to the percentage of identity) is represented above the sequences. The limits of the exons are indicated by the grey bars. Alignments were obtained by MUSCLE and submitted to the GUIDANCE filter (more details in the “Methods” section). The regions of the alignments that were not well supported by the filter were removed (shaded areas) from further phylogenetic analyses. Bsy: Branchipolynoe symmytilida, Bse: B. seepensis, D: domain, SD: single-domain. [file 40064_2015_1124_MOESM3_ESM.png]

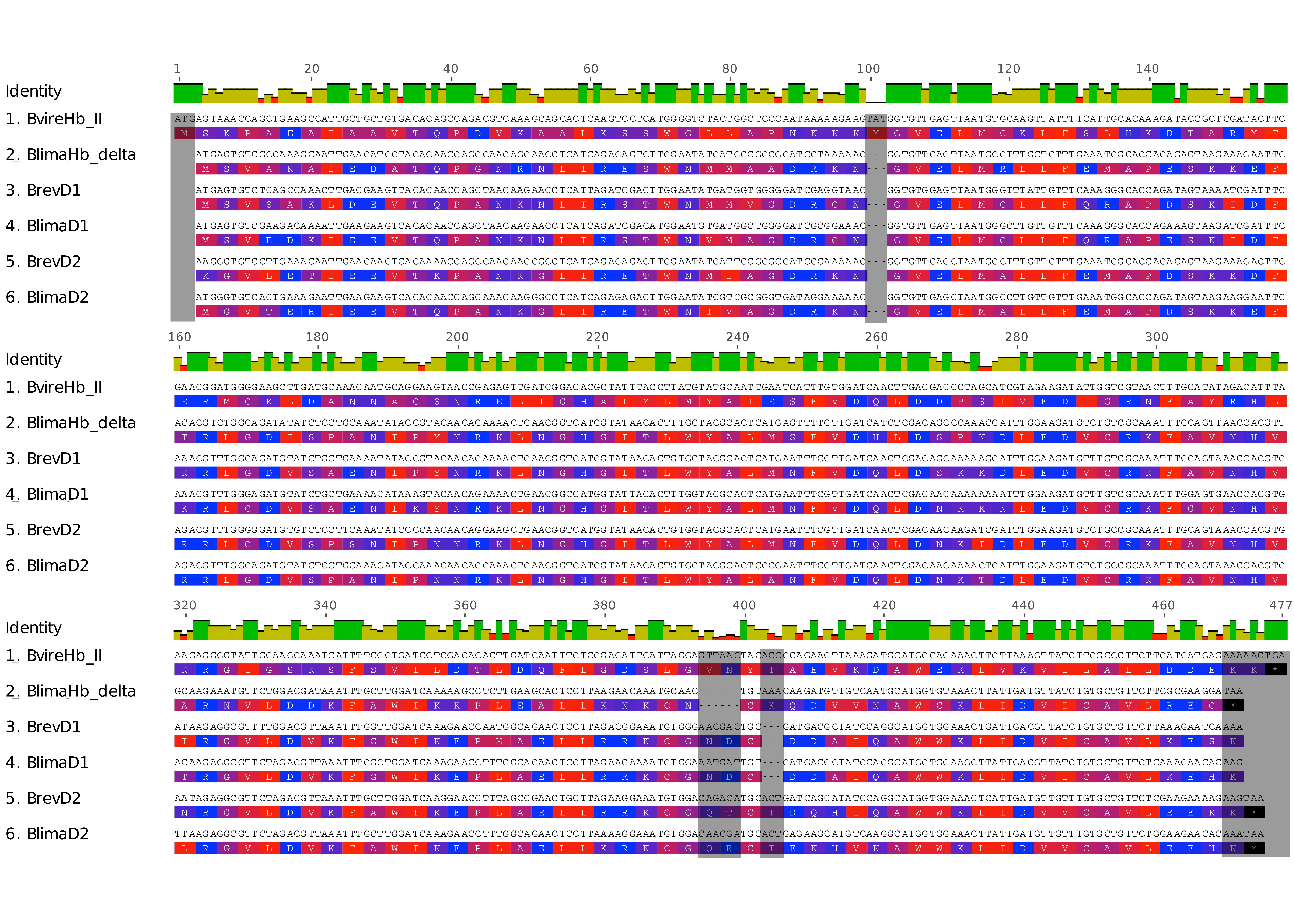

Supplement: Additional file 4: — Figure S3. Alignment of the single-domain and the di-domain globin sequences from the mollusk species. Overall sequence identity (red = low identity, green = high identity, the height of blocks is proportional to the percentage of identity) is represented above the sequences. Please refer to the legend in Figure S2 for the color codes in nucleotide identity and residue hydrophobicity. These sequences were generated from mRNA and for this reason exon limits are not depicted. Alignments were obtained by MUSCLE and submitted to the GUIDANCE filter (more details in the “Methods” section). The regions of the alignments that were not well supported by the filter were removed (shaded areas) from further phylogenetic analyses. Bvire: Barbatia virecens, Blima: Barbatia lima, Brev: B. reveeana, Hb: hemoglobin, D: domain. [file 40064_2015_1124_MOESM4_ESM.png]
