# Supplementary material for: Selective forces acting during multi-domain protein evolution: the case of multi-domain globins
Source: Springerplus. 2015 Jul 16;4:354. doi: 10.1186/s40064-015-1124-2 (PMC4503718; doi:10.1186/s40064-015-1124-2)
Supplement: Additional file 2: — Table S1. Identity between orthologs domains (amino acid sequences) from the three analyzed groups of multidomain invertebrate Hbs. [file 40064_2015_1124_MOESM2_ESM.docx]

Table S1. Identity between orthologs domains (amino acid sequences) from the three analyzed groups of multidomain invertebrate Hbs.

|  |  |  |  |  | References |
| --- | --- | --- | --- | --- | --- |
|  | BseD1 | BseD2 | BseD3 | BseD4 |  |
| BsyD1 | 97.7% |  |  |  | this publication |
| BsyD2 |  | 94% |  |  | “ |
| BsyD3 |  |  | 97.7% |  | “ |
| BsyD4 |  |  |  | 94% | “ |
|  | PdecD1 | PdecD2 |  |  |  |
| AsuumD1 | 73% |  |  |  | Gibson et al. 1993 |
| AsuumD2 |  | 51% |  |  | “ |
|  | BlimaD1 | BlimaD2 |  |  |  |
| BreevD1 | 91% |  |  |  | Suzuki and Arita 1995 |
| BreevD2 |  | 88% |  |  | “ |
